# The effectiveness of coronavirus disease 2019 (COVID-19) vaccine in the prevention of post–COVID-19 conditions: A systematic literature review and meta-analysis

**DOI:** 10.1017/ash.2022.336

**Published:** 2022-12-06

**Authors:** Alexandre R. Marra, Takaaki Kobayashi, Hiroyuki Suzuki, Mohammed Alsuhaibani, Shinya Hasegawa, Joseph Tholany, Eli Perencevich, Aline Miho Maezato, Victoria Catharina Volpe Ricardo, Jorge L. Salinas, Michael B. Edmond, Luiz Vicente Rizzo

**Affiliations:** 1 Department of Internal Medicine, University of Iowa Carver College of Medicine, Iowa City, Iowa, United States; 2 Faculdade Israelita de Ciências da Saúde Albert Einstein, Hospital Israelita Albert Einstein, São Paulo, SP, Brazil; 3 Center for Access & Delivery Research & Evaluation (CADRE), Iowa City Veterans’ Affairs Health Care System, Iowa City, Iowa, United States; 4 Department of Pediatrics, King Faisal Specialist Hospital & Research Centre, Riyadh, Saudi Arabia; 5 Stanford University, Stanford, California, United States; 6 West Virginia University School of Medicine, Morgantown, West Virginia, United States

## Abstract

**Background:**

Although multiple studies have revealed that coronavirus disease 2019 (COVID-19) vaccines can reduce COVID-19–related outcomes, little is known about their impact on post–COVID-19 conditions. We performed a systematic literature review and meta-analysis on the effectiveness of COVID-19 vaccination against post–COVID-19 conditions (ie, long COVID).

**Methods:**

We searched PubMed, CINAHL, EMBASE, Cochrane Central Register of Controlled Trials, Scopus, and Web of Science from December 1, 2019, to April 27, 2022, for studies evaluating COVID-19 vaccine effectiveness against post–COVID-19 conditions among individuals who received at least 1 dose of Pfizer/BioNTech, Moderna, AstraZeneca, or Janssen vaccine. A post–COVID-19 condition was defined as any symptom that was present 3 or more weeks after having COVID-19. Editorials, commentaries, reviews, study protocols, and studies in the pediatric population were excluded. We calculated the pooled diagnostic odds ratios (DORs) for post–COVID-19 conditions between vaccinated and unvaccinated individuals. Vaccine effectiveness was estimated as 100% × (1 − DOR).

**Results:**

In total, 10 studies with 1,600,830 individuals evaluated the effect of vaccination on post–COVID-19 conditions, of which 6 studies were included in the meta-analysis. The pooled DOR for post–COVID-19 conditions among individuals vaccinated with at least 1 dose was 0.708 (95% confidence interval (CI), 0.692–0.725) with an estimated vaccine effectiveness of 29.2% (95% CI, 27.5%–30.8%). The vaccine effectiveness was 35.3% (95% CI, 32.3%–38.1%) among those who received the COVID-19 vaccine before having COVID-19, and 27.4% (95% CI, 25.4%–29.3%) among those who received it after having COVID-19.

**Conclusions:**

COVID-19 vaccination both before and after having COVID-19 significantly decreased post–COVID-19 conditions for the circulating variants during the study period although vaccine effectiveness was low.

An estimated >200 million people have been affected globally by the long-term effects of coronavirus disease 2019 (COVID-19), known as post–COVID-19 conditions (also known as long COVID).^1^ In the third year of the pandemic, individuals are still at risk of acquiring COVID-19, even with authorized vaccines available.^2,3^

A growing body of early global research shows that the authorized COVID-19 vaccines remain highly protective against multiple outcomes including asymptomatic infection, hospitalization, reinfection, and death.^4–8^ Vaccine effectiveness is a measure of how well vaccination protects individuals against outcomes.^3,4,9,10^ Vaccine effectiveness differs from the efficacy measured in a trial because the efficacy cannot predict exactly how effective vaccination will be for a much bigger and more variable population being vaccinated in more real-life conditions.^11^ Although vaccine effectiveness after 2 doses of Pfizer/BioNTech vaccine against COVID-19 caused by the original SARS-CoV-2 variant was reported to be >95%,^7^ the primary immunization provided limited protection against the newer variants.^12^ The boosters substantially increased protection; however, that protection is known to wane over time.^13^

Whether vaccination reduces the risk of post–COVID-19 is currently unknown, and few studies have assessed vaccine effectiveness against post–COVID-19 conditions.^14,15^ The Centers for Disease Control and Prevention (CDC) defines post–COVID-19 conditions (ie, long COVID) as a vast range of ongoing health problems (eg, cardiovascular, respiratory, and neuropsychiatric symptoms) that can last for >4 weeks after an individual has been infected by SARS-CoV-2 virus.^16^ We reviewed the literature on the effectiveness of COVID-19 vaccines for post–COVID-19 conditions, and we pooled the results of published studies to allow for more precise effectiveness estimates.

## Methods

### Systematic literature review and inclusion and exclusion criteria

This review was conducted according to the Preferred Reporting Items for Systematic Reviews and Meta-Analysis (PRISMA) statement^17^ and the Meta-analysis of Observational Studies in Epidemiology (MOOSE) guidelines^18^ and was registered on Prospero (https://www.crd.york.ac.uk/PROSPERO/) on March 17, 2022 (registration no. CRD42022318686). Approval by the institutional review board was not required. We applied the following inclusion criteria: original research manuscript; published in peer-reviewed, scientific journals; involved vaccinated and unvaccinated individuals; evaluated the long-term effectiveness of the COVID-19 vaccine; and observational study design. Post–COVID-19 conditions were defined as a wide range of health symptoms that are present 3 or more weeks after having COVID-19.^16^ The literature search included studies from December 1, 2019, to April 27, 2022. Editorials, commentaries, reviews, study protocols, and studies in the pediatric population were excluded. Studies without comparison between vaccinated and unvaccinated individuals (or other vaccinated control group) were also excluded.

### Search strategy

**Fig. 1.**
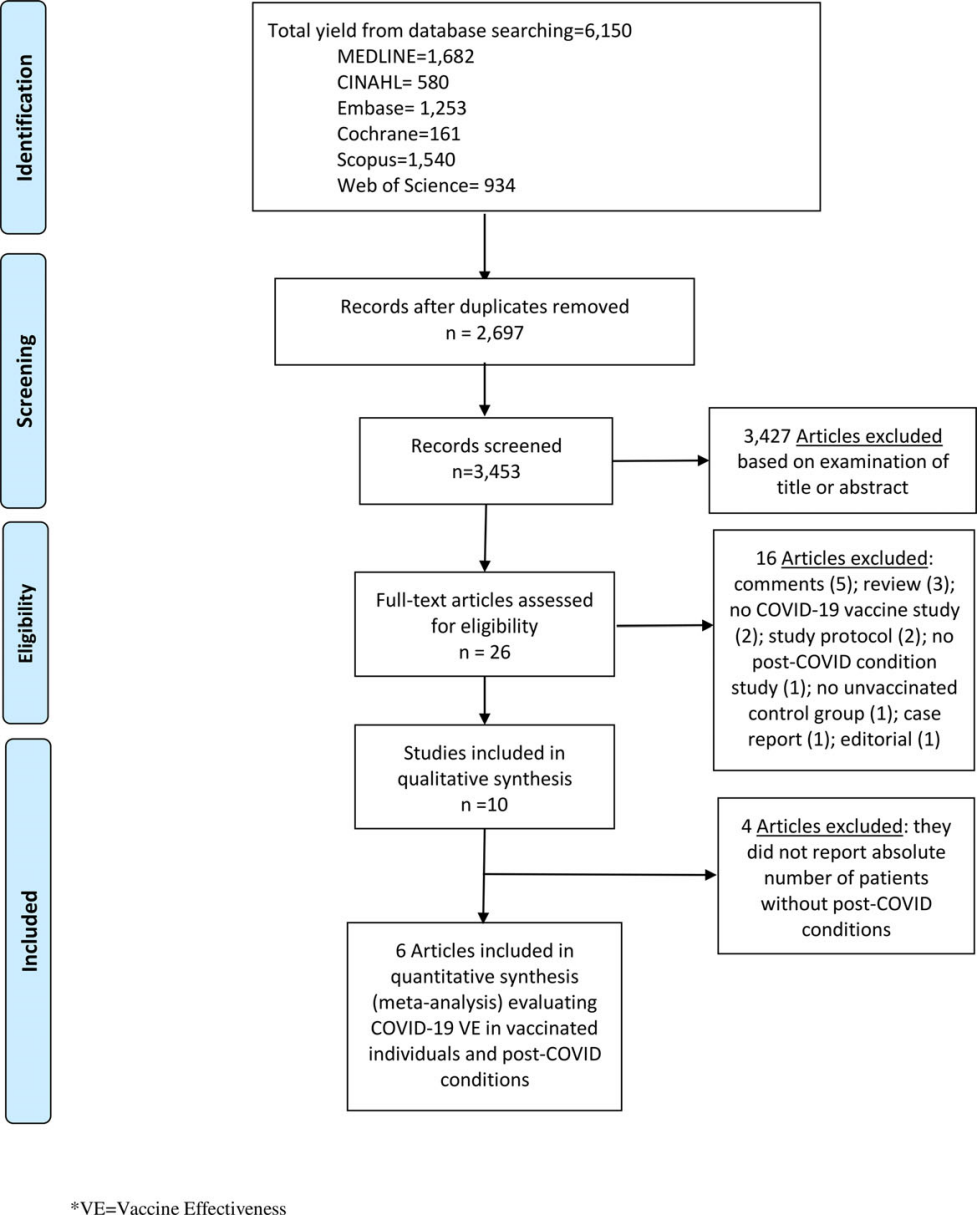
Literature search for articles on the COVID-19 vaccine effectiveness in post–COVID-19 conditions.

We performed literature searches in PubMed, Cumulative Index to Nursing and Allied Health (CINAHL), Embase (Elsevier Platform), Cochrane Central Register of Controlled Trials, Scopus (which includes EMBASE abstracts), and Web of Science. The entire search strategy is described in Supplementary Appendix 1. We reviewed the reference lists of retrieved articles to identify studies that were not identified from the preliminary literature searches. After applying exclusion criteria, we reviewed 26 papers, 10 of which met the inclusion criteria and were included in the systematic literature review (Fig. 1).

### Data abstraction and quality assessment

Titles and abstracts of all articles were screened to assess whether they met inclusion criteria. Abstract screening was performed by 1 reviewer (A.R.M.). Among the 8 independent reviewers (A.M.M., A.R.M., H.S., J.T., M.A., S.H., T.K., and V.C.V.R.), 2 reviewers abstracted data for each article. Reviewers resolved disagreements by consensus.

The reviewers abstracted data on study design, population and location, study period (months) and calendar time, demographic and characteristics of participants, and types of COVID-19 vaccine (if available). Post–COVID-19 conditions were considered the primary outcome to calculate vaccine effectiveness after at least 1 dose of a COVID-19 vaccine. Risk of bias was assessed using the Downs and Black scale.^19^ Reviewers answered all questions from this scale as written except for question number 27 (ie, a single item on the power subscale scored 0–5), which was changed to a yes or no. Two authors performed component quality analysis independently, reviewed all inconsistent assessments, and resolved disagreements by consensus.^20^

### Statistical analysis

To perform a meta-analysis on the extracted data, we calculated the pooled diagnostic odds ratio (DORs) for post–COVID-19 conditions between vaccinated (received at least 1 dose of a COVID-19 vaccine) and unvaccinated individuals. Vaccine effectiveness was estimated as 100% × (1 − DOR). We performed stratified analyses by the timing of the COVID-19 vaccine: those with COVID-19 vaccines before or after COVID-19 diagnosis, those with COVID-19 vaccines after COVID-19 diagnosis.^21–26^ We performed statistical analysis using R version 4.1.0 software (R Foundation for Statistical Computing, Vienna, Austria) with mada package version 0.5.4.^27^ Analogous to the meta-analysis of the odds ratio methods for the DOR, an estimator of random-effects model following the approach of DerSimonian and Laird is provided by the mada package.^27^ For our meta-analysis of vaccine effectiveness estimates against post–COVID-19 conditions, we used a bivariate random-effects model, adopting a similar concept of performing the diagnostic accuracy. This enabled simultaneous pooling of sensitivity and specificity with mixed-effect linear modeling while allowing for the trade-off between them.^28,29^ Heterogeneity between studies was evaluated using I^2^ estimation and the Cochran Q statistic test.

## Results

### Characteristics of included studies

In total, 10 studies met the inclusion criteria^21–26,30–33^ and were included in the final review (Table 1). All studies were nonrandomized; of these, 5 were prospective cohort studies,^22,23,26,32,33^ 4 were retrospective cohort studies,^24,25,30,31^ and 1 was a case–control study.^21^ Of these 10 studies, 9 studies evaluated the Pfizer/BioNTech vaccine.^21–26,30,32,33^ Also, 7 studies analyzed the Moderna vaccine,^21,22,24,25,30,32,33^ 6 studies analyzed the Janssen vaccine,^22,24,25,30,32,33^ and 5 studies analyzed the AstraZeneca vaccine.^21,22,26,30,32^

**Table 1.** Summary of Characteristics of Studies Included in the Systematic Literature Review

First Author, Year, Location, Study Design, Study Period (month) and Dates	COVID-19 Vaccine and COVID-19 Vaccine Before COVID-19 (Yes or No)	No. of Participants and Characteristics	Post–COVID-19 Condition		Post–COVID-19 Condition		Post–COVID-19 Condition Definition	Symptoms Included in Post–COVID-19 Condition Studies	Benefit of COVID-19 Vaccines to Decrease post–COVID-19 Symptoms	D&B Score (max, 28)
			Vaccinated Second Dose	Control Group (Unvaccinated)	Vaccinated First Dose	Control Group (Unvaccinated)				
Antonelli 2021, UK^24^ Case-control study 7 (Dec 2020–Jul 2021)	Pfizer/BioNTech, Moderna, and AstraZeneca Yes	1,240,009 participants (COVID Symptoms Study app users) reported first vaccine dose (6,030 (0.5% tested positive for SARS-CoV-2), and 971,504 reported second dose (2,370 (0.2% tested positive for SARS-CoV-2)	2,370 (NR absolute numbers of long COVID in vaccinated group)	2,370 (NR absolute numbers of long COVID in unvaccinated group)	906 (31 of 592 vaccinated with long COVID)	906 (55 of 427 unvaccinated with long COVID)	Long duration of COVID-19 symptoms ≥28 d	-General: fatigue, post-exertional malaise, fever -Respiratory and hearth: shortness of breath, cough, chest pain, fast-beating or pounding heart -Neurological: difficult thinking or concentrating (“brain fog”), headache, mood changes, change in smell or taste, dizziness or lightheadedness, pins-and-needles feelings -Digestive: abdominal pain, diarrhea -Other: joint or muscle pain	Yes. Compared with unvaccinated individuals, after their second dose of COVID-19 vaccine, individuals were less likely to have prolonged illness (symptoms ≥28 days).	21
Arnold 2021, UK^29^ Prospective cohort study 10 (Apr 2020–Jan 2021)	Pfizer/BioNTech, and AstraZeneca No	66 participants (44 vaccinated matched with 22 unvaccinated (Most were highly symptomatic of long COVID at 8 months (82% in both groups had at least 1 persistent symptom, with fatigue, shortness of breath, and insomnia predominanting)	NR	NR	44 (36 with long COVID)	22 (18 with long COVID)	NR	-General: fatigue -Respiratory and hearth: shortness of breath, cough, chest pain, fast-beating or pounding heart -Neurological: difficult thinking or concentrating (“brain fog”), headache, change in smell or taste, sleep problems, pins-and-needles feelings -Digestive: abdominal pain, diarrhea -Other: joint or muscle pain	No. COVID-19 vaccination was not associated in a worsening of symptoms or quality of life in patients with long COVID.	18
Kuodi 2022, Israel^26^ Prospective cohort study 20 (March 2020–Nov 2021)	Pfizer/BioNTech Yes	3,388 participants (951 with COVID-19 (340 (36%) reported receiving 1 dose of COVID-19 vaccine, and 294 (31%) reported 2 doses) * vs 2,437 without COVID-19	294 (167 with long COVID)	317 (217 with long COVID)	340 (252 with long COVID)	317 (217 with long COVID)	WHO definition: “A condition which occurs in individuals with a history of probable or -Confirmed severe acute respiratory syndrome coronavirus 2 (SARS-CoV-2) infection -Usually 3 months from the onset of COVID-19 with -Symptoms that last for at least 2 months and cannot be explained by an alternative diagnosis.”	-General: fatigue, post-exertional malaise -Respiratory and heart: shortness of breath, chest pain, fast-beating or pounding heart -Neurological: difficult thinking or concentrating (“brain fog”), headache, change in smell or taste, dizziness or lightheadedness, pins-and-needles feelings -Digestive: abdominal pain, diarrhea -Other: joint or muscle pain	Yes. Vaccination with at least 2 doses of COVID-19 vaccine reduced the most common symptoms (eg, fatigue, headache, and muscle pain).	21
Peghin 2022, Italy^25^ Prospective cohort study 15 (March 2021–May, 2021)	Pfizer/BioNTech, Moderna, Janssen, and AstraZeneca No	479 participants [132 vaccinated with 1 dose and 111 with 2 doses (all mRNA COVID-19 vaccine) vs 347 unvaccinated with previous COVID-19]	NR	NR	132 (44 with long COVID)	347 (157 with long COVID)	Long duration of COVID-19 symptoms ≥12 weeks	-General: fatigue -Respiratory and heart: shortness of breath, cough, chest pain -Neurological: headache, mood changes, change in smell or taste, dizziness or lightheadedness -Digestive: abdominal pain, diarrhea -Other: joint or muscle pain, rash	No. However, COVID-19 vaccination was not associated with the emergence of post–COVID-19 symptoms over 1 year after acute infection.	21
Scherlinger,2021, France^33^ Retrospective cohort study 0.5 (Aug 3–17, 2021)	Pfizer/BioNTech, Moderna, Janssen, and AstraZeneca Yes (and after having COVID-19)	567 participants in an online questionnaire (397 vaccinated with 1 dose and 170 unvaccinated)	NR	NR	397 (397 with long COVID)	170 (170 with long COVID)	Long duration of COVID-19 symptoms ≥4 weeks	General: fatigue, fever -Respiratory and heart: shortness of breath, cough, chest pain, fast-beating or pounding heart -Neurological: difficult thinking or concentrating (“brain fog”), headache, mood changes,change in smell or taste, sleep problems, pins-and-needles feeling -Digestive: abdominal pain, diarrhea -Other: joint or muscle pain	No. However, COVID-19 vaccination was well tolerated in most long COVID patients.	19
Selvaskandan, 2022, UK^34^ Retrospective cohort study 1 (Mar 31, 2021–May 1, 2021)	NR Unknown	423 participants (online questionnaire at the UK Nephrology Work Force)	340 of 363 (86%) received first dose (NR absolute numbers of long COVID in vaccinated group)	21 (NR absolute numbers of long COVID in unvaccinated group)	398 of 419 (95%) received first dose (NR absolute numbers of long COVID in vaccinated group)	21 (NR absolute numbers of long COVID in unvaccinated group)	Long duration of COVID-19 symptoms ≥12 weeks	-General: fatigue, postexertional malais -Respiratory and heart: shortness of breath, cough, fast-beating or pounding heart -Neurological: difficult thinking or concentrating (“brain fog”), headache, mood changes, change in smell or taste, sleep problems -Other: joint or muscle pain	NR	13
Simon, 2021, USA^28^ Retrospective cohort study 15 (Feb 2020–May 2021)	Pfizer/BioNTech, Moderna, and Janssen Yes (and after having COVID-19)	240,648 participants with COVID-19 (2,392 vaccinated with 1 dose of COVID-19 vaccine prior to the diagnosis vs 220,460 unvaccinated prior to 12 weeks after their COVID-19 diagnosis)	NR	NR	2,392 (382 with long COVID)	220,460 (84,408 with long COVID)	Long-COVID cases were classified as those in which the patient presented 1 or more COVID-19–associated symptoms between 12 and 20 weeks after having COVID-19. ICD-10 codes were used detect the post–COVID-19 conditions.	NR	Yes. COVID-19 vaccination is protective against long COVID.	21
Taquet, 2022, UK^27^ Retrospective cohort study 8 (Jan 1, 2021–Aug 31, 2021)	Pfizer/BioNTech, Moderna, and Janssen Yes	10,024 participants with COVID-19 recorded at least 2 weeks after a first dose of COVID-19 vaccine vs 83,957 unvaccinated (after propensity scored matching: 9,479 were matched to unvaccinated participants)	6,957 (4,459 with long COVID)	6,957 (4,459 with long COVID)	2,996 (1,908 with long COVID)	2,996 (1,953 with long COVID)	6-month incidence of all long-COVID outcomes. ICD-10 codes were used detect the post–COVID-19 conditions.	-General: fatigue -Respiratory and heart: shortness of breath, chest pain -Neurological: difficult thinking or concentrating (“brain fog”), headache, mood changes, sleep problems, pins-and-needles feelings -Digestive: abdominal pain -Other: joint or muscle pain	No. Receiving at least 1 dose of COVID-19 vaccine was not associated with a lower risk of long COVID.	21
Tran, 2021, France^35^ Prospective cohort study 22 (Nov 2020–Sept 2021)	Pfizer/BioNTech, Moderna, Janssen, and AstraZeneca No	910 participants (455 vaccinated with 1 dose of COVID-19 vaccine vs 455 unvaccinated participants)	NR	NR	455 (NR absolute numbers of long COVID in vaccinated group)	455 (NR absolute numbers of long COVID in unvaccinated group)	-Participants with confirmed or suspected COVID-19 -Infection and symptoms persisting >3 weeks past the initial infection and who reported at least 1 symptom attributable to long COVID-19 at baseline	-Long COVID ST score (range, 0–53), evaluating the severity of long COVID at 120 days after baseline -Long COVID IT score (range, 0–60) evaluating the impact of long COVID at 120 days after baseline -A pair of validated patient-reported instruments assessing respectively 53 long-COVID symptoms -6 dimensions of patients’ lives that can be affected by COVID-19	Yes. COVID-19 vaccination reduced the life impact of long COVID among patients with persistent symptoms.	21
Wisnivesky, 2022, USA^36^ Prospective cohort study 7 (Jul 2020–Feb 2021)	Pfizer/BioNTech, Moderna, and Janssen No	453 COVID-19 participants (324 vaccinated with 1 dose and 129 unvaccinated)	NR	NR	324 (324 with long COVID)	129 (129 with long COVID)	Participants who reported at least 1 long-COVID symptom at baseline and 6-month follow-up interview	-General: fatigue -Respiratory and heart: shortness of breath, cough -Neurological: difficult thinking or concentrating (“brain fog”), headache, change in smell or taste, sleep problems	No. COVID-19 vaccination was not associated with improvement in post–COVID-19 conditions.	21

Note. D&B score, Downs & Black score; ICD-10, *International Classification of Diseases, Tenth Revision*; WHO, World Health Organization; NR, not reported.

* At the time of data collection, very few individuals had received a third dose, and those who did were recorded as 2 doses.

Of the 10 studies included in our review, 4 were conducted in the United Kingdom.^21,24,26,31^ Also, 2 studies were performed in the United States,^25,33^ 2 studies were performed in France,^30,32^ and 1 study was performed in Israel^23^ and in Italy.^22^ All studies were performed between March 2020 and November 2021.^21–26,30–33^ The study duration varied from 2 weeks to 22 months.^21–26,30–33^

In our qualitative analysis, 10 studies including 1,600,830 individuals evaluated the effect of vaccination on post–COVID-19 conditions.^21–26,30–33^ Moreover, 4 studies evaluated vaccine effectiveness among those who received the COVID-19 vaccine only after having COVID-19,^22,26,32,33^ 3 studies evaluated vaccine effectiveness for post–COVID-19 conditions among those who were vaccinated before having COVID-19,^21,23,24^ 2 studies evaluated vaccine effectiveness among those who were vaccinated before and after COVID-19,^25,30^ and 1 study evaluated vaccine effectiveness but did not specify the timing of the vaccine.^31^ All 10 studies evaluated at least 1 dose of a COVID-19 vaccine,^21–26,30–33^ and 4 studies evaluated vaccinated individuals with 2 doses vaccine.^21,23,24,31^ None of these studies reported genomic surveillance data when evaluating the post–COVID-19 conditions in either vaccinated or unvaccinated individuals.^21–26,30–33^

Each study adopted different definitions for post–COVID-19 conditions (Table 1). Post–COVID-19 conditions were defined as symptoms lasting >12 weeks in 3 studies,^22,23,31^ >4 weeks in 2 studies,^21,30^ >6 months in 2 studies,^24,33^ and >3 weeks in 1 study.^32^ One study defined symptoms between 12 and 20 weeks as a post–COVID-19 condition,^25^ and 1 study did not report the duration of symptoms.^26^ All studies used at least 1 of the common symptoms (details shown in Table 1) to make a diagnosis of a post–COVID-19 condition.^21–26,30–33^ Half of the included studies (5 studies)^22,24,26,30,33^ did not report any benefit of COVID-19 vaccination in reducing post–COVID-19 condition symptoms. Also, 4 studies showed that vaccination was protective against post–COVID-19 symptoms,^21,23,25,32^ and 1 study did not report any statistical analysis of effectiveness.^31^

**Fig. 2.**
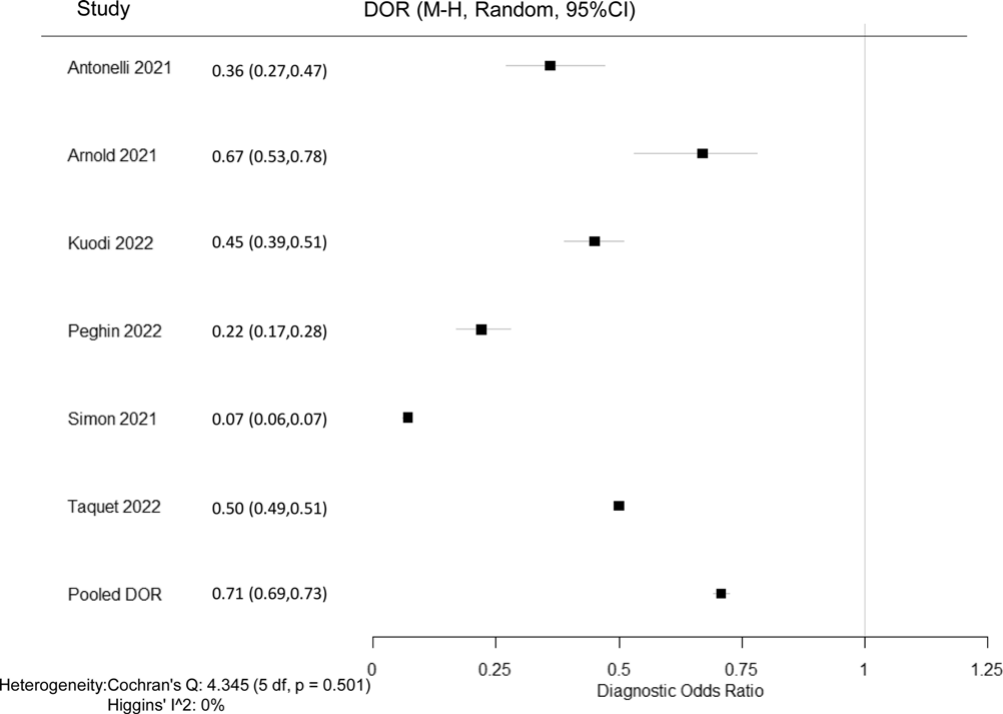
Forest plot of COVID-19 vaccine effectiveness among post–COVID-19 conditions in individuals who received COVID-19 vaccine before or after having COVID-19. Diagnostic odds ratios (DOR) were determined with the Mantel-Haenszel random-effects method. Note. CI, confidence interval; M-H, Mantel-Haenszel.

Overall, 6 studies including 251,123 individuals evaluated post–COVID-19 conditions among those who received COVID-19 vaccine before or after having COVID-19 (Table 2) and were included in the meta-analysis.^21–26^ The pooled prevalence of post–COVID-19 conditions was 39.1% among those who were unvaccinated and 37.6% among those who received at least 1 dose. The pooled DOR for post–COVID-19 conditions among individuals vaccinated with at least 1 dose was 0.708 (95% CI, 0.692–0.725) with an estimated vaccine effectiveness of 29.2% (95% CI, 27.5%–30.8%) (Fig. 2). Of the 6 studies, 4 evaluated post–COVID-19 conditions in individuals who received the COVID-19 vaccine only before infection.^21,23–25^ The DOR was 0.647 (95% CI, 0.619–0.677) (Supplementary Appendix 2), and the estimated vaccine effectiveness was 35.3% (95% CI, 32.3%–38.1%) (Table 2). Only 3 papers evaluated post–COVID-19 conditions for those who received the vaccine only after infection.^22,25,26^ The DOR was 0.726 (95% CI, 0.707–0.746) (Supplementary Appendix 3), and the estimated vaccine effectiveness was 27.4% (95% CI, 25.4%–29.3%) (Table 2). Because only 2 papers evaluated post–COVID-19 conditions for those who received 2 doses,^23,24^ we did not perform a stratified analysis. The results of meta-analyses were homogeneous for studies evaluating post–COVID-19 conditions in individuals who received the COVID-19 vaccine before or after having COVID-19 (heterogeneity *P* = .50; I^2^ = 0%). The results were homogenous for studies evaluating post–COVID-19 conditions in individuals who received a vaccine before infection (heterogeneity *P* = .68; I^2^ = 0%). The results were also homogenous for studies evaluating post–COVID-19 conditions in individuals who received a vaccine after infection (heterogeneity *P* = .62; I^2^ = 0%), respectively.

**Table 2.** Subset Analyses Evaluating COVID-19 Vaccine Effectiveness Among post–COVID-19 Condition in Individuals Who Received COVID-19 Vaccine Before or After Having COVID-19

COVID-19 vaccine before/after having COVID-19^a^	Studies Included, No.	Participants, No.	Pooled Diagnostic Odds Ratio (DOR) (95% CI)	I^2^ Test for Heterogeneity	Vaccine Effectiveness, % (95% CI) ^b^
Before/After^24–29^	6	251,123	0.708 (0.692–0.725)	0%	29.2 (27.5–30.8)
Before^24,26–28^	4	250,578	0.647 (0.619–0.677)	0%	35.3 (32.3–38.1)
After^25,28,29^	3	223,397	0.726 (0.707–0.746)	0%	27.4 (25.4–29.3)

Note. CI, confidence interval.

a There is overlapping (vaccine effectiveness for post–COVID-19 condition who got COVID-19 vaccine before and after having COVID-19) in 1 of the studies.^28^

b Vaccine Effectiveness was estimated as 100% × (1 − DOR).

Regarding the quality assessment scores of the 10 included studies, 8 studies were considered good quality (ie, 19–23 of 28 possible points) according to the Downs and Black quality tool.^21–25,30,32,33^ One study was considered fair (14–18 points),^26^ and another study was considered poor quality (≤13 points).^31^

## Discussion

This systematic literature review and meta-analysis indicated that the pooled prevalence of post–COVID-19 conditions was 39.1% among those unvaccinated and 37.6% among those vaccinated at least once. The vaccine effectiveness of at least 1 dose of COVID-19 vaccines (primarily the mRNA vaccines) against post–COVID-19 conditions was low at ∼30%; however, the prevalence of post–COVID-19 conditions was lower, with statistically significant difference in vaccinated individuals. Given that the stratified analysis showed a significant reduction of post–COVID-19 conditions with the vaccine even after having COVID-19, vaccine should be offered to unvaccinated individuals who have had COVID-19.

With the ongoing COVID-19 pandemic, a considerable proportion of people who have recovered from COVID-19 have long-term symptoms involving multiple organs and systems.^34,35^ A recent systematic review and meta-analysis demonstrated that long COVID is a public health issue with a global estimated pooled prevalence of 43% (95% CI, 39%–46%) and that hospitalized, and nonhospitalized patients have an estimated pooled prevalence of 54% (95% CI, 44%–63%) and 34% (95% CI, 25%–46%), respectively.^1^ Another systematic review including 57 studies reported that more than half of COVID-19 survivors experienced persistent post–COVID-19 condition symptoms 6 months after recovery.^35^ Our systematic review showed a relatively low prevalence of post–COVID-19 conditions; this is likely because most individuals included in our studies were nonhospitalized individuals. According to a prior study, the prevalence is highest in Asia (51%), followed by Europe (44%), and North America (31%)^1^.

The studies included in our systematic review used a variety of symptoms and durations to make a diagnosis of post–COVID-19 conditions. The most common symptoms described were fatigue or muscle weakness, persistent muscle pain, anxiety, memory problems, sleep problems, and shortness of breath.^1,34,35^ Another study reported that, regardless of the initial disease severity, COVID-19 survivors had longitudinal improvements in physical and mental health, with most returning to their original work within 2 years.^34^ However, survivors had a remarkably lower health status than the general population at 2 years.^34^ The CDC reports that individuals with post–COVID-19 conditions may experience many symptoms that can last >4 weeks or even months after infection and the symptoms may initially resolve but subsequently recur.^16^ This finding differs from the World Health Organization (WHO) definition in which post–COVID-19 conditions are defined to occur in individuals who have a history of probable or confirmed SARS-CoV-2 infection, usually within 3 months from the onset of COVID-19, with symptoms and effects that last for at least 2 months.^36^ Two studies in our systematic literature review used *International Classification of Diseases, Tenth Revision* (ICD-10) codes to detect post–COVID-19 conditions.^24,25^ We do not believe that ICD codes are accurate enough to detect most post–COVID-19 conditions, and they would not capture severity. A clearer and more standardized definition of post–COVID-19 conditions is needed for researchers to investigate the true prevalence among those who are vaccinated and unvaccinated and to evaluate the vaccine effectiveness against post–COVID-19 conditions.

Our meta-analysis demonstrated that vaccine effectiveness among those who received the vaccine before COVID-19 was 35%, although vaccine effectiveness among those who received the vaccine after infection was 27%, suggesting that protection against post–COVID-19 conditions due to vaccine is more effective if a vaccine is given before infection. The studies included in our systematic literature review helped us to better understand the vaccine effectiveness against post–COVID-19 conditions in the context of a global pandemic with new SARS-CoV-2 variants^9,10^ and to better understand that COVID-19 vaccination was significantly associated with lower post–COVID-19 conditions, even among those who received a COVID-19 vaccine after infection. Although some patients who previously contracted COVID-19 are hesitant to get vaccinated,^37^ our findings can reassure that individuals with prolonged COVID-19 symptoms who have not been vaccinated that they should do so.^22,25^

Our study had several limitations. First, most of the included studies in the meta-analysis were observational studies, which are subject to multiple biases.^38^ However, this is the most common study design in the infection prevention literature.^38^ Second, we could not perform further analyses about possible adverse events after vaccination. Only 1 of the included studies reported possible adverse events after vaccine administration.^32^ In contrast, receiving COVID-19 vaccines was not associated with a worsening of symptoms in patients with post–COVID-19 conditions.^25^ Also, 1 of the included studies measured SARS-CoV-2 antibodies assessing 2 different serological assays to distinguish between response to vaccination (receptor-binding domain-RBD SARS-CoV-2 IgG) and/or natural infection (non-RBD- SARS-CoV-2 IgG)^22^ and reported that there were no significant differences in the worsening of post–COVID-19 symptoms (22.7% vs 15.8%; *P* = .209) between vaccinated and unvaccinated individuals.^22^ In addition, the persistence of high serological titer response induced by natural infection but not by vaccination may play a role in post–COVID-19 conditions.^22^ Third, we could not perform further analyses stratified by immunocompromised status due to the limited number of studies. None of the studies compared immunocompromised to immunocompetent individuals. Fourth, since our study focused on vaccine effectiveness against post–COVID-19 conditions after at least 1 COVID-19 vaccine dose, we could not evaluate the impact of a booster dose on post–COVID-19 condition vaccine effectiveness. Only 1 study reported patients receiving a third dose of COVID-19 vaccine, but that study did not include further analysis.^23^ Fifth, because of the low number of included studies in our meta-analysis, it was not possible to perform a stratified analysis by vaccine type (ie, mRNA, viral vector or inactivated virus vaccines) or to evaluate their effect on post–COVID-19 conditions. Only 1 study evaluated a single-vaccine Pfizer/BioNTech vaccine,^23^ the other studies used aggregated data of any other COVID-19 vaccine (ie, Pfizer/BioNTech, or Moderna or Janssen, and/or AstraZeneca), considering just 1 or 2 doses of those COVID-19 vaccines for the analysis. We included individuals in the meta-analysis who received at least 1 dose of COVID-19 vaccine because only 2 of the included studies reported data after receiving 2 doses.^23,24^ It was not possible to evaluate vaccine effectiveness against each of the symptoms of post–COVID-19 because no data were reported for each of the vaccination status groups (vaccinated and unvaccinated individuals). Therefore, we decided to perform our meta-analysis and stratified analysis using a bivariate approach to preserve the two-dimensional nature of the original data from the selected studies.^21–26^ Thus, the results of our meta-analysis should be interpreted with caution, particularly because only a few studies were included, and one of these studies had >200,000 individuals in the sample.^25^ Additionally, it was not possible to control for other confounding (eg, age) and pre-existing conditions. Only 1 study performed a matched case–control study adjusted for age, body mass index, and sex among the 4 studies that included a meta-analysis evaluated post–COVID-19 conditions in individuals who received the COVID-19 vaccine before they had COVID-19.^21^ Lastly, because the definitions of post–COVID-19 conditions varies significantly among the included studies, overdiagnosis and misdiagnosis could have affected the reported results.

In conclusion, COVID-19 vaccination before and after having COVID-19 provided a low but statistically significant decrease in post–COVID-19 conditions for the variants circulating during the study period. To better understand vaccine effectiveness against post–COVID-19 conditions, more observational studies are needed to evaluate other types of COVID-19 vaccines (eg, inactivated virus), vaccination after having COVID-19, vaccine effectiveness of a booster dose, vaccine effectiveness of mixing COVID-19 vaccines, and genomic surveillance. A more standardized definition of post–COVID-19 conditions is also needed both for research and clinical purposes.

## Supplementary material

For supplementary material accompanying this paper visit https://doi.org/10.1017/ash.2022.336. click here to view supplementary material

## References

[1] Chen C , Haupert SR , Zimmermann L , Shi X , Fritsche LG , Mukherjee B. Global prevalence of post COVID-19 condition or long COVID: a meta-analysis and systematic review. J Infect Dis 2022;226:1593–1607. 35429399 10.1093/infdis/jiac136 PMC9047189

[2] Brown CM , Vostok J , Johnson H , et al. Outbreak of SARS-CoV-2 infections, including COVID-19 vaccine breakthrough infections, associated with large public gatherings—Barnstable County, Massachusetts, July 2021. Morbid Mortal Wkly Rep 2021;70:1059–1062. 10.15585/mmwr.mm7031e2 PMC8367314 34351882

[3] Glatman-Freedman A , Hershkovitz Y , Kaufman Z , Dichtiar R , Keinan-Boker L , Bromberg M. Effectiveness of BNT162b2 vaccine in adolescents during outbreak of SARS-CoV-2 delta variant infection, Israel, 2021. Emerg Infect Dis 2021;27:2919–2922. 34570694 10.3201/eid2711.211886 PMC8544958

[4] Chodick G , Tene L , Rotem RS , et al. The effectiveness of the two-dose BNT162b2 vaccine: analysis of real-world data. Clin Infect Dis 2022;74:472–478. 33999127 10.1093/cid/ciab438 PMC8240867

[5] Del Rio C , Malani PN , Omer SB. Confronting the delta variant of SARS-CoV-2, summer 2021. JAMA 2021;326:1001–1002. 34406361 10.1001/jama.2021.14811

[6] Levin-Rector A , Firestein L , McGibbon E , et al. Reduced odds of SARS-CoV-2 reinfection after vaccination among New York City adults, July–November 2021. *Clin Infect Dis* 2022. doi: 10.1093/cid/ciac380. 10.1093/cid/ciac380 PMC9129172 35594552

[7] Polack FP , Thomas SJ , Kitchin N , et al. Safety and efficacy of the BNT162b2 mRNA COVID-19 vaccine. N Engl J Med 2020;383:2603–2615. 33301246 10.1056/NEJMoa2034577 PMC7745181

[8] Baden LR , El Sahly HM , Essink B , et al. Efficacy and safety of the mRNA-1273 SARS-CoV-2 vaccine. N Engl J Med 2021;384:403–416. 33378609 10.1056/NEJMoa2035389 PMC7787219

[9] Lopez Bernal J , Andrews N , Gower C , et al. Effectiveness of COVID-19 vaccines against the B.1.617.2 (delta) variant. N Engl J Med 2021;385:585–594. 34289274 10.1056/NEJMoa2108891 PMC8314739

[10] Lopez Bernal J , Andrews N , Gower C , et al. Effectiveness of the Pfizer-BioNTech and Oxford-AstraZeneca vaccines on COVID-19 related symptoms, hospital admissions, and mortality in older adults in England: test negative case-control study. BMJ (Clin Res) 2021;373:n1088. 10.1136/bmj.n1088 PMC8116636 33985964

[11] Vaccine efficacy, effectiveness and protection. World Health Organization website. https://www.who.int/news-room/feature-stories/detail/vaccine-efficacy-effectiveness-and-protection. Published 2021. Accessed October 13, 2022.

[12] Collie S , Champion J , Moultrie H , Bekker LG , Gray G. Effectiveness of BNT162b2 vaccine against omicron variant in South Africa. N Engl J Med 2022;386:494–496. 34965358 10.1056/NEJMc2119270 PMC8757569

[13] Andrews N , Stowe J , Kirsebom F , et al. COVID-19 vaccine effectiveness against the omicron (B.1.1.529) variant. N Engl J Med 2022;386:1532–1546. 35249272 10.1056/NEJMoa2119451 PMC8908811

[14] Arnold DT , Milne A , Samms E , Stadon L , Maskell NA , Hamilton FW. Symptoms After COVID-19 vaccination in patients with persistent symptoms after acute infection: a case series. Ann Intern Med 2021;174:1334–1336. 34029484 10.7326/M21-1976 PMC8252827

[15] Venkatesan P. Do vaccines protect from long COVID? Lancet Respir Med 2022;10:e30. 35065716 10.1016/S2213-2600(22)00020-0 PMC8776281

[16] National Center for Immunization and Respiratory Diseases (NCIRD) Division of Viral Diseases. Long COVID or post-COVID conditions. Centers for Disease Control and Prevention website.https://www.cdc.gov/coronavirus/2019-ncov/long-term-effects/index.html. Published 2022. Accessed May 20, 2022.

[17] Moher D , Liberati A , Tetzlaff J , Altman DG. Preferred reporting items for systematic reviews and meta-analyses: the PRISMA statement. PLoS Med 2009;6:e1000097. 19621072 10.1371/journal.pmed.1000097 PMC2707599

[18] Stroup DF , Berlin JA , Morton SC , et al. Meta-analysis of observational studies in epidemiology: a proposal for reporting. Meta-analysis Of Observational Studies in Epidemiology (MOOSE) group. JAMA 2000;283:2008–2012. 10789670 10.1001/jama.283.15.2008

[19] Downs SH , Black N. The feasibility of creating a checklist for the assessment of the methodological quality both of randomised and non-randomised studies of health care interventions. J Epidemiol Commun Health 1998;52:377–384. 10.1136/jech.52.6.377 PMC1756728 9764259

[20] Alderson PGS HJ, editors. Assessment of study quality. In: Cochrane Reviewer’s Handbook version 4.2.3. Chichester, UK: John Wiley & Sons; 2004.

[21] Antonelli M , Penfold RS , Merino J , et al. Risk factors and disease profile of post-vaccination SARS-CoV-2 infection in UK users of the COVID Symptom Study app: a prospective, community-based, nested, case-control study. Lancet Infect Dis 2022;22:43–55. 34480857 10.1016/S1473-3099(21)00460-6 PMC8409907

[22] Peghin M , De Martino M , Palese A , et al. Post–COVID-19 syndrome and humoral response association after one year in vaccinated and unvaccinated patients. Clin Microbiol Infect 2022;28:1397–1398. 35842022 10.1016/j.cmi.2022.06.003 PMC9279256

[23] Kuodi P , Gorelik Y , Zayyad H , et al. Association between vaccination status and reported incidence of post-acute COVID-19 symptoms in Israel: a cross-sectional study of patients infected between March 2020 and November 2021. NPJ Vaccines 2022;7:101. 36028498 10.1038/s41541-022-00526-5 PMC9411827

[24] Taquet M , Dercon Q , Harrison PJ. Six-month sequelae of post-vaccination SARS-CoV-2 infection: a retrospective cohort study of 10,024 breakthrough infections. Brain Behav Immun 2022;103:154–162. 35447302 10.1016/j.bbi.2022.04.013 PMC9013695

[25] Simon MA , Luginbuhl RD , Parker R. Reduced incidence of long-COVID symptoms related to administration of COVID-19 vaccines both before COVID-19 diagnosis and up to 12 weeks after. *medRxiv* 2021 doi: 10.1101/2021.11.17.21263608.

[26] Arnold D , Milne A , Samms E , Stadon L , Maskell N , Hamilton F. Are vaccines safe in patients with Long COVID? A prospective observational study. *medRxiv* 2021. doi: 10.1101/2021.03.11.21253225.

[27] Doebler P , Holling H , Sousa-Pinto B. Meta-analysis of diagnostic accuracy with mada. R Project Organization website. https://cran.r-project.org/web/packages/mada/vignettes/mada.pdf. Published 2017. Accessed November 21, 2022.

[28] Reitsma JB , Glas AS , Rutjes AW , Scholten RJ , Bossuyt PM , Zwinderman AH. Bivariate analysis of sensitivity and specificity produces informative summary measures in diagnostic reviews. J Clin Epidemiol 2005;58:982–990. 16168343 10.1016/j.jclinepi.2005.02.022

[29] Goto M , Ohl ME , Schweizer ML , Perencevich EN. Accuracy of administrative code data for the surveillance of healthcare-associated infections: a systematic review and meta-analysis. Clin Infect Dis 2014;58:688–696. 24218103 10.1093/cid/cit737

[30] Scherlinger M , Pijnenburg L , Chatelus E , et al. Effect of SARS-CoV-2 vaccination on symptoms from post-acute sequelae of COVID-19: results from the nationwide VAXILONG study. Vaccines (Basel) 2021;10:46. 10.3390/vaccines10010046 PMC8781023 35062706

[31] Selvaskandan H , Nimmo A , Savino M , et al. Burnout and long COVID among the UK nephrology workforce: results from a national survey investigating the impact of COVID-19 on working lives. Clin Kidney J 2022;15:517–526. 35198158 10.1093/ckj/sfab264 PMC8754810

[32] Tran V-T , Perrodeau É , Saldanha J , Pane I , Ravaud P. Efficacy of COVID-19 vaccination on the symptoms of patients with long COVID: a target trial emulation using data from the ComPaRe e-cohort in France. 2022. 10.1136/bmjmed-2022-000229 PMC9978748 36910458

[33] Wisnivesky JP , Govindarajulu U , Bagiella E , et al. Association of vaccination with the persistence of post-COVID symptoms. J Gen Intern Med 2022;37:1748–1753. 35266128 10.1007/s11606-022-07465-w PMC8906626

[34] Huang L , Li X , Gu X , et al. Health outcomes in people 2 years after surviving hospitalisation with COVID-19: a longitudinal cohort study. Lancet Respir Med 2022;10:863–876. 35568052 10.1016/S2213-2600(22)00126-6 PMC9094732

[35] Groff D , Sun A , Ssentongo AE , et al. Short-term and long-term rates of postacute sequelae of SARS-CoV-2 infection: a systematic review. JAMA Network Open 2021;4:e2128568. 34643720 10.1001/jamanetworkopen.2021.28568 PMC8515212

[36] Coronavirus disease (COVID-19): post–COVID-19 condition. World Health Organization website. https://www.who.int/news-room/questions-and-answers/item/coronavirus-disease-(covid-19)-post-covid-19-condition. Accessed May 21, 2022.

[37] Gerussi V , Peghin M , Palese A , et al. Vaccine hesitancy among Italian patients recovered from COVID-19 infection towards influenza and SARS-Cov-2 vaccination. Vaccines 2021;9:172. 33670661 10.3390/vaccines9020172 PMC7922251

[38] Harris AD , Lautenbach E , Perencevich E. A systematic review of quasi-experimental study designs in the fields of infection control and antibiotic resistance. Clin Infect Dis 2005;41:77–82. 15937766 10.1086/430713

