# Supplementary material for: The effectiveness of coronavirus disease 2019 (COVID-19) vaccine in the prevention of post–COVID-19 conditions: A systematic literature review and meta-analysis
Source: Antimicrob Steward Healthc Epidemiol. 2022 Dec 6;2(1):e192. doi: 10.1017/ash.2022.336 (PMC9726631; doi:10.1017/ash.2022.336)
Supplement: Supplementary file 1 [file ashsup.zip › S2732494X22003369sup003.docx]

**Supplementary Appendix 1.** Search terms and strategies

| Covid 19 | Vaccine efficacy | Long term symptoms |
| --- | --- | --- |
| MeSH  COVID-19  SARS-CoV-2  Coronavirus Infections (no explode) | MeSH  Vaccine efficacy  Covid-19 Vaccines  RNA, Messenger  Vaccines  Immunization (inc vaccination)  **AND**  Treatment outcome | MeSH  Complications subheading  Post acute covid 19 syndrome  Chronic disease |
| Keywords  Covid  Covid 19  Corona virus  Coronavirus  2019-nCoV  SARS-CoV-2  2019-nCoV | Keywords  Vaccine(s)  Vaccination(s)  Vaccinated  Pfizer  Bnt162b2  Moderna  immunization(s)  variolation(s)  Immunologic stimulation  Immunostimulation  Immunotherap (y,ies)  mRNA-1273  ChAdOx1-S  AZD1222  AstraZeneca/Oxford  Janssen  Johnson&Johnson  Ad26COVS1  JNJ-78436735  Gamaleya  Sputnik V  Sinovac  Corona Vac  Novavax  Covaxin  sinopharm  **AND**  Effectiveness  Efficacy  Effective  Antibody response  Antibody levels  IgG  Neutralizing antibodies  Success | Keywords  Long  hauler  Long term  Long haul  Post acute  Chronic  Expanded  extended  recurr*  sustain*  persist*  prolong*  continu*  Debilitating  **AND**  effect*  symptom*  impact*  outcome*  recover*  suffer*  sequela*  impair* |

**MEDLINE via Ovid 4/27/22**

**
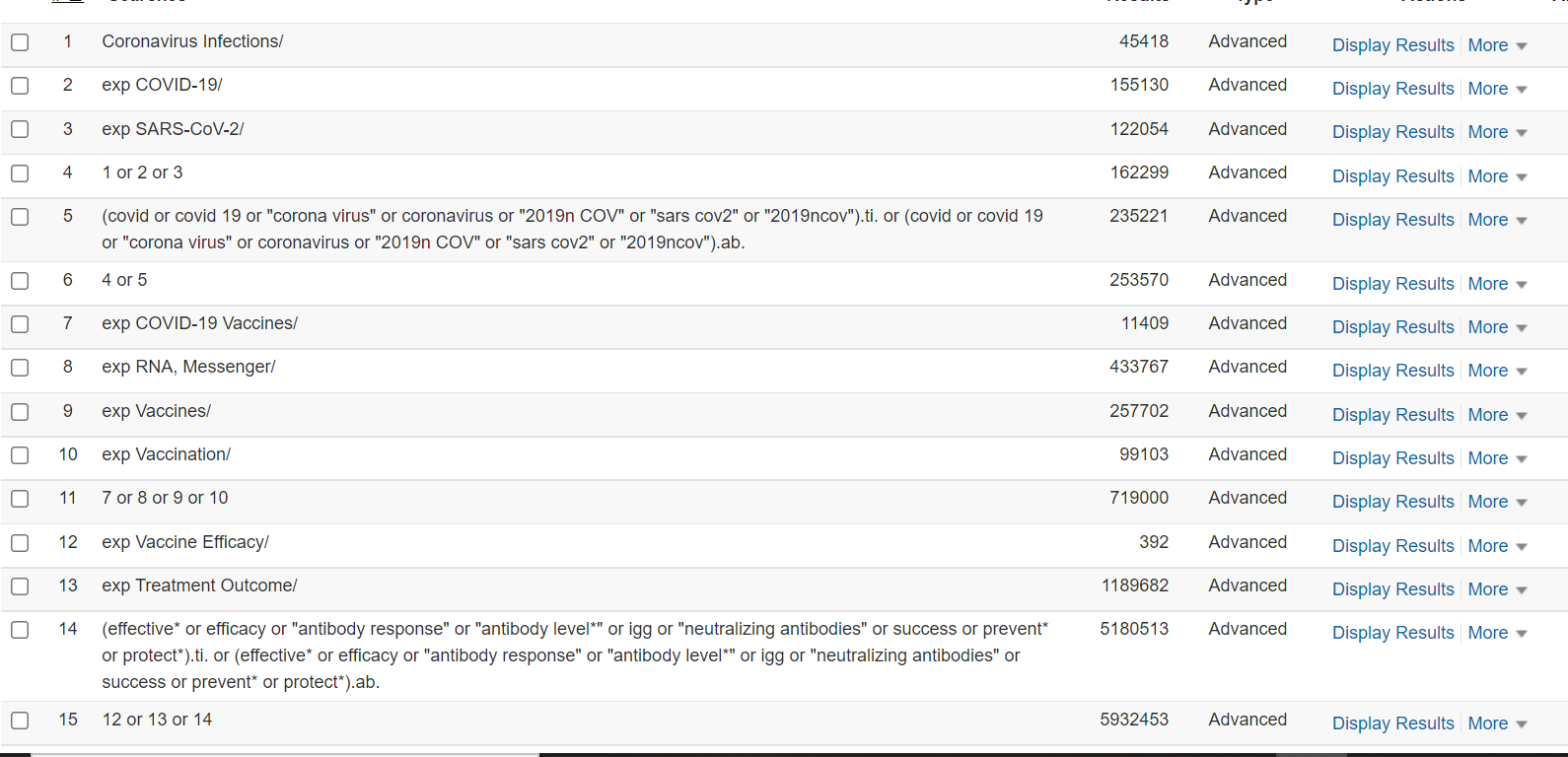
**

**
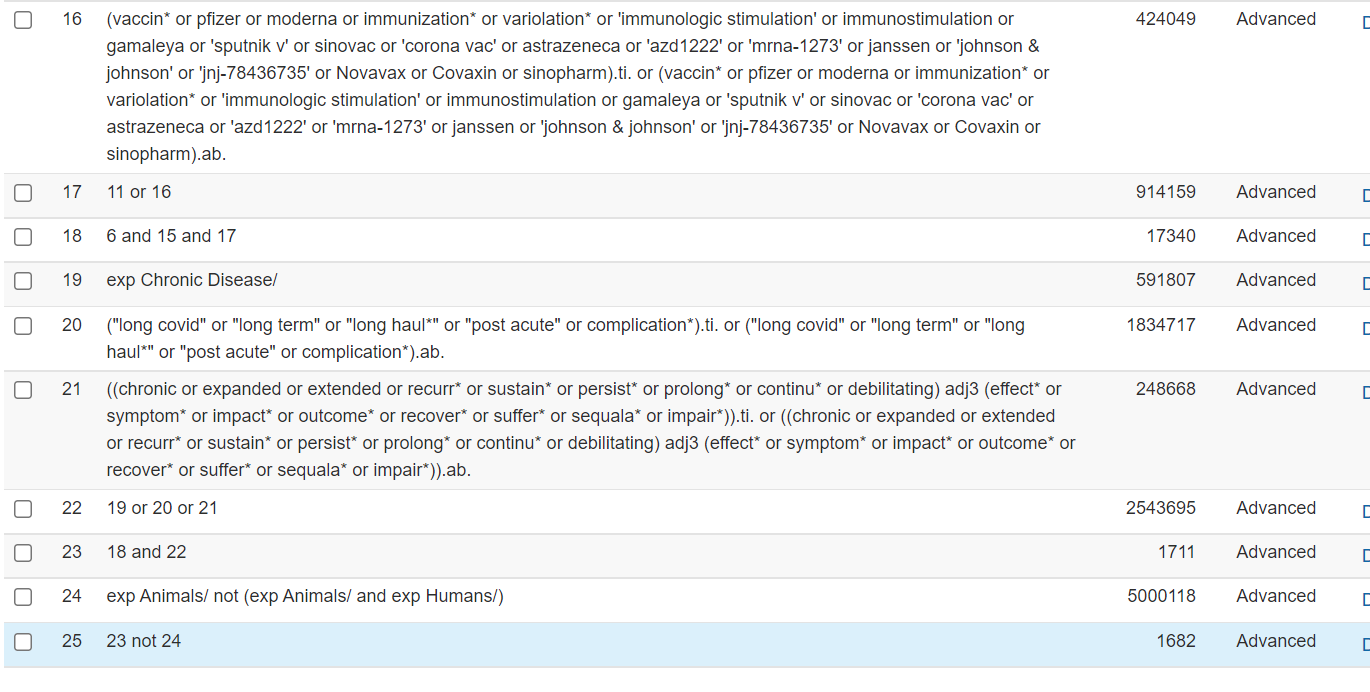
**

**Embase 4/27/22**

#1

'long covid'/exp

OR

'long haul':ab,ti OR 'long hauler':ab,ti OR 'long term':ab,ti OR 'long covid':ab,ti OR 'post acute':ab,ti

OR

((chronic OR expanded OR extended OR recurr* OR sustain* OR persist* OR prolong* OR continu* OR debilitating) NEAR/2 (effect* OR symptom* OR impact* OR outcome* OR recover* OR suffer* OR sequala* OR impair*)):ti,ab

#2

'coronavirus disease 2019'/exp OR 'coronavirus infection'/de OR covid:ab,ti OR 'covid 19':ab,ti OR 'corona virus':ab,ti OR coronavirus:ab,ti OR 'sars-cov-2':ab,ti OR '2019-ncov':ab,ti

#3

'messenger rna'/exp OR 'vaccine'/exp OR 'vaccination'/exp OR 'immunization'/exp OR vaccine*:ab,ti OR vaccination*:ab,ti OR pfizer:ab,ti OR moderna:ab,ti OR immunization*:ab,ti OR variolation*:ab,ti OR 'immunologic stimulation':ab,ti OR immunostimulation:ab,ti OR gamaleya:ab,ti OR 'sputnik v':ab,ti OR sinovac:ab,ti OR 'corona vac':ab,ti OR astrazeneca:ab,ti OR 'azd1222':ab,ti OR 'mrna-1273':ab,ti OR janssen:ab,ti OR 'johnson & johnson':ab,ti OR 'jnj-78436735':ab,ti OR Novavax:ab,ti OR Covaxin:ab,ti OR sinopharm:ab,ti

#4

'drug efficacy'/exp OR 'treatment outcome'/exp OR effectiveness:ti,ab OR efficacy:ti,ab OR effective:ti,ab OR 'antibody response':ab,ti OR 'antibody levels':ab,ti OR igg:ab,ti OR 'neutralizing antibodies':ab,ti OR success:ti,ab OR prevent:ti,ab OR prevention:ti,ab OR protect:ti,ab OR protection:ti,ab OR protecting:ti,ab OR 'sars-cov-2 antibody'/exp

#1 AND #2 AND #3 AND #4=1253

**CINAHL 4/27/22**

#1

(MH "RNA, Messenger" OR MH "Vaccines+" OR MH "Immunization" OR Vaccine* OR Vaccination* OR  Pfizer OR  Moderna OR immunization* OR variolation* OR “immunologic stimulation" OR  Immunostimulation OR Gamaleya OR "Sputnik V" OR  Sinovac OR "Corona Vac" OR AstraZeneca OR Janssen OR "AZD1222" OR "mRNA-1273" OR Janssen OR "Johnson & Johnson" OR "JNJ-78436735") AND (MH "Coronavirus Infections" OR MH "COVID-19" OR MH "SARS-CoV-2" OR Covid OR "Covid 19" OR "Corona virus" OR Coronavirus OR "2019-nCoV" OR "SARS-CoV-2" OR   "2019-nCoV")

OR

MH "COVID-19 Vaccines"

#2

"long haul" OR "long hauler" OR "long term" OR "long covid" OR "post acute"

OR

(chronic OR expanded OR extended OR recurr* OR sustain* OR persist* OR prolong* OR continu* OR debilitating) N2 (effect* OR symptom* OR impact* OR outcome* OR recover* OR suffer* OR sequala* OR impair*)

#3

(MH "Drug Efficacy") OR (MH "Treatment Outcomes+")

OR

TI ( Effective* OR efficacy OR “antibody response'” OR “antibody levels” OR igg OR “neutralizing antibodies” OR success OR prevent OR prevention OR protect* OR “sars-cov-2 antibody” ) OR AB ( Effective* OR efficacy OR “antibody response'” OR “antibody levels” OR igg OR “neutralizing antibodies” OR success OR prevent OR prevention OR protect* OR “sars-cov-2 antibody” )

#1 AND #2 AND #3=580

**Scopus 4/27/22**

#1

TITLE-ABS-KEY ( vaccine* OR  vaccination* OR pfizer OR moderna OR immunization* OR  variolation* OR  "immunologic stimulation"OR immunostimulation  OR  gamaleya  OR  "Sputnik V"  OR  sinovac  OR  "Corona Vac"  OR  astrazeneca  OR  janssen  OR  "AZD1222"  OR  "mRNA-1273" OR janssen OR "Johnson & Johnson" OR "JNJ-78436735") AND  TITLE-ABS-KEY ( covid OR "Covid 19" OR "Corona virus" OR coronavirus OR 2019ncov OR  sarscov2 OR  2019-ncov)

#2

(chronic OR expanded OR extended OR recurr* OR sustain* OR persist* OR prolong* OR  continu* OR  debilitating) W/2 (effect* OR symptom* OR  impact* OR outcome* OR recover*  OR suffer* OR sequala* OR  impair*)

OR

TITLE-ABS-KEY "long haul" OR "long hauler" OR "long term" OR "long covid" OR "post acute"

#3

 TITLE (effective* OR efficacy OR "antibody response'" OR "antibody levels" OR igg OR  "neutralizing antibodies" OR success OR prevent OR prevention OR protect* OR "sars-cov-2 antibody") OR ABS (effective* OR efficacy OR "antibody response'" OR "antibody levels" OR  igg OR "neutralizing antibodies" OR success OR prevent OR prevention OR protect* OR  "sars-cov-2 antibody")

#1 AND #2 AND #3=1540, without reviews=1068

**Web of Science 4/27/22**

TS=(vaccine* OR vaccination*OR pfizer OR moderna OR immunization* OR  variolation* OR  "immunologic stimulation"OR immunostimulation  OR  gamaleya  OR  "Sputnik V" OR sinovac OR "Corona Vac" OR astrazeneca OR janssen OR "AZD1222"  OR "mRNA-1273" OR janssen OR "Johnson & Johnson" OR "JNJ-78436735") AND TS=(covid OR "Covid 19" OR "Corona virus" OR coronavirus OR 2019ncov OR  sarscov2 OR  2019-ncov)

#2

TS=(chronic OR expanded OR extended OR recurr* OR sustain* OR persist* OR prolong* OR  continu* OR  debilitating) NEAR/2 (effect* OR symptom* OR  impact* OR outcome* OR recover*  OR suffer* OR sequala* OR  impair*)

OR

TS=("long haul" OR "long hauler" OR "long term" OR "long covid" OR "post acute" )

#3

TI=(effective* OR efficacy OR "antibody response'" OR "antibody levels" OR igg OR  "neutralizing antibodies" OR success OR prevent OR prevention OR protect* OR "sars-cov-2 antibody") OR AB=(effective* OR efficacy OR "antibody response'" OR "antibody levels" OR igg OR  "neutralizing antibodies" OR success OR prevent OR prevention OR protect* OR "sars-cov-2 antibody")

#1 AND #2 AND #3=934

**Cochrane 4/27/22**

**
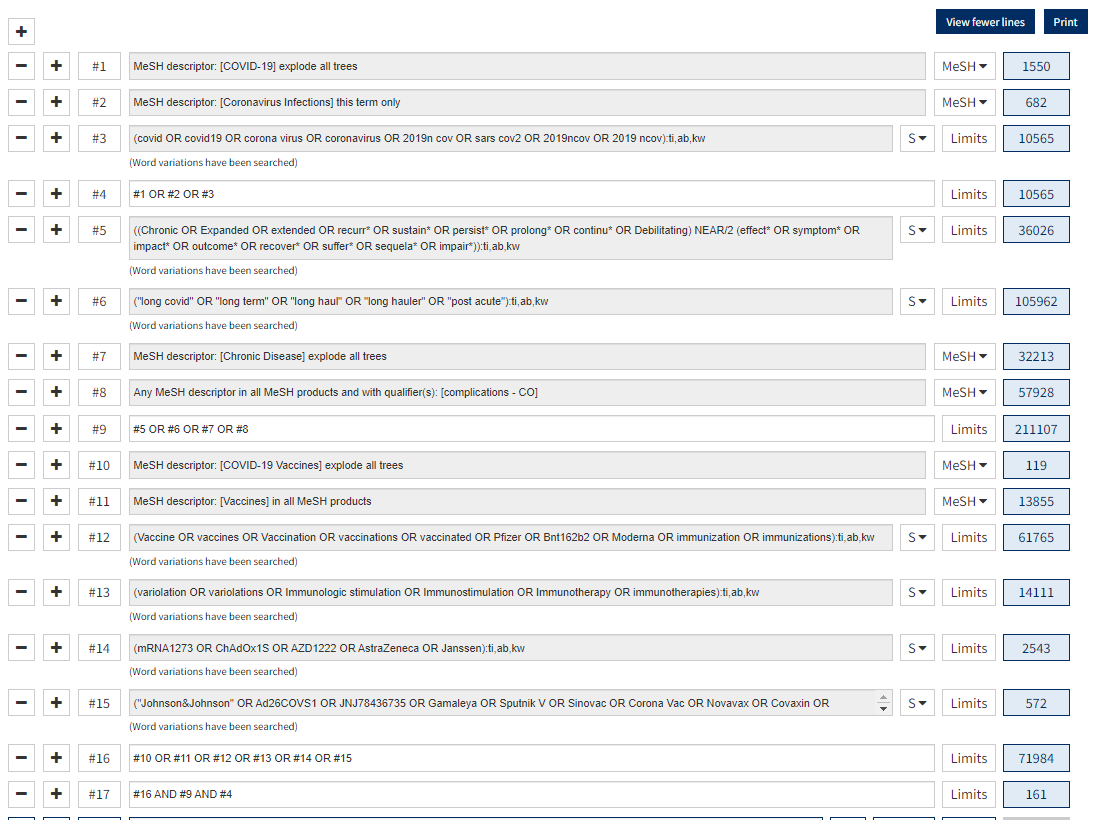
**
